# Hemoadsorption to treat severe iatrogenic intoxication with Patent Blue: a case report

**DOI:** 10.1186/s13256-020-02657-6

**Published:** 2021-02-09

**Authors:** Fabio Silvio Taccone, Mickael Gardette, Jacques Creteur, Alexandre Brasseur, Sophie Lorent, David Grimaldi

**Affiliations:** 1 Department of Intensive Care Erasme Hospital, Free University of Brussels, Route de Lennik 808, 1070 Brussels, Belgium; 2 Department of Pharmacy, Free University of Brussels, Brussels, Belgium

**Keywords:** Intoxication, Multiple organ failure, Methamphetamine, Patent Blue, Hemoadsorption, Case report

## Abstract

**Background:**

Intoxication with Patent Blue V [sodium compound of (diethylamino-4-phenyl)(hydroxy-5-disulfo-2,4-phenyl) methanol] can lead to high levels of methemoglobin and metabolic acidosis. In severe cases and if not rapidly eliminated from the plasma, this can lead to multiple organ failure and death.

**Case report:**

A 27-year-old Asian woman (original from Vietnam) was admitted after ecstasy intoxication resulting in multi-organ failure (acute respiratory distress syndrome, metabolic acidosis, capillary leakage syndrome, renal failure, shock refractory to standard resuscitation). As a consequence, continuous renal replacement therapy and veno-venous extracorporeal membrane oxygenation were started. Methylene blue administration to reverse vasoplegia was decided, but unfortunately, Patent Blue V was erroneously administered, resulting in a severe clinical picture of methemoglobinemia and tissue hypoxia. As a therapeutic intervention, CytoSorb hemoadsorption was initiated, and rapid and significant reduction in plasma methemoglobin, accompanied by improved hemodynamics and normalization in plasma lactate levels, was observed.

**Conclusions:**

This is the first case describing the application of CytoSorb hemoadsorption in a patient with ecstasy intoxication complicated by iatrogenic administration of Patent Blue V. There is a potential role for CytoSorb in drug intoxication, which needs to be confirmed in larger series.

## Background

Since the late 1980s, the synthetic amphetamine derivative 3.4-methylenedioxymethamphetamine (MDMA or ecstasy) has gained substantial popularity as a recreational drug, particularly among young party-goers at electronic dance music venues. Individuals who take MDMA describe a sense of euphoria, wakefulness, intimacy, sexual arousal, and disinhibition [1]. Initially developed in 1914 as an appetite inhibitor but never approved for marketing, MDMA is primarily used for recreational purposes, and the potential for abuse was quickly recognized. Its neurochemical effects are comparable to amphetamines and methamphetamines and include stimulation of catecholamine release (that is, norepinephrine and dopamine) and blockage of their presynaptic re-uptake. Moreover, this drug also enhances both serotonergic and dopaminergic release, as well as inhibition of serotonin re-uptake [2]. The widespread popularity of ecstasy is based on the misguided assumption that this drug is associated only with little potential toxicity. However, ecstasy has addictive psychoactive properties, and its abuse has led to an alarming increase of admissions to emergency departments worldwide. Indeed, complications are frequent and range from minor side-effects, such as bruxism, tachycardia, and trismus, midweek “lows” and a prolonged “hangover,” to severe and potentially life-threatening complications including hyperpyrexia, rhabdomyolysis, multiple organ failure, disseminated intravascular coagulation (DIC), serotonin syndrome, acute panic disorder, hyponatremia with cerebral edema, and sudden cardiac death [3]. MDMA may also occasionally have an impact on systemic hemodynamics, triggering capillary leakage syndrome and shock states, which are refractory to standard resuscitation. In this context, methylene blue (that is methylthioninium chloride) may be a potential treatment for refractory shock, as it prevents nitric oxide-mediated vasodilation and contributes to maintaining adequate organ perfusion [4].

Another medically used dye is Patent Blue V, that is, sodium compound of (diethylamino-4-phenyl)(hydroxy-5-disulfo-2,4-phenyl) methanol, which is routinely used in lymphangiography and sentinel node biopsy as a dye to color lymph vessels. In some cases, its application can lead to skin sensitivity, rash, itching, and nausea but also urticaria, hypotension, and bronchospasm [5]. In the circulation, the dye is highly protein-bound to albumin, excreted in the urine within 24–48 hours but also into the bile, and exerts an inhibitory effects on the mitochondrial function of human cells. Markers for intoxication with Patent Blue are significantly increased plasma levels of methemoglobin (metHb) and metabolic acidosis. In severe cases and if not rapidly eliminated from the plasma, this can lead to multiple organ failure and death.

A new hemoadsorption device named CytoSorb, initially intended for the treatment of critically illness associated with elevated cytokine levels (that is, sepsis, after cardiac surgery), has been recently proven to effectively reduce circulating levels of several medications for which no valid antidote is available (that is, ticagrelor and rivaroxaban) [6–8]. The adsorption process is dependent on hydrophobic interactions and molecular size, and is also concentration dependent. In the absence of effective measures to quickly reduce toxic plasma levels of Patent Blue, CytoSorb could serve as a potential therapy.

We report herein a case of severe MDMA intoxication resulting in capillary leakage syndrome and shock refractory to standard resuscitation. Instead of methylene blue administration, Patent Blue V was erroneously administered to counteract shock, resulting in a severe clinical picture of methemoglobinemia and tissue hypoxia, which was successfully treated by CytoSorb hemoadsorption.

## Case presentation

A previously healthy 27-year-old Asian woman (original from Vietnam) (body weight 45 kg) was admitted to the emergency department of a peripheral hospital in Brussels due to intoxication of unknown origin. The patient had no past medical history, no previous pregnancies, and a degree in economic sciences, and had been working in an economic consulting agency since 3 years previously; she had no chronic medical therapy prior to this intoxication, did not smoke, and rarely consumed alcohol. Also, no particular medical history from her family was reported. On the day of admission, she had been in a nightclub and was found on the floor with rigor, tachycardia, hypertension (190/100 mmHg), hyperthermia (41.6 °C), agitation, and altered consciousness. Direct intubation was performed; blood and urine drug screening checked positive for 3.4-methylendioxy-*N*-methylamphetamine without concomitant alcohol intake. Upon admission to the emergency department, she was sedated and paralyzed and had dilated pupils; no abnormalities were found on lung, abdominal, cutaneous, and joint examination. No other focal neurological signs were observed (that is, asymmetric reflexes or pyramidal syndrome). Heart rate was 130 bpm, blood pressure dropped to 75/35 mmHg, and body temperature increased to 41.8 °C. She showed no signs of cyanosis or hypoperfusion. Initial echocardiography showed a left ventricular ejection fraction of 50%; chest X-ray revealed bilateral infiltrates (probably due to aspiration), while electrocardiogram showed sinus tachycardia. Initial blood gas indicated the following: pH 7.28, PaCO_2_ 32 mmHg, PaO_2_ 182 mmHg (on FiO_2_ 100%), lactate 3.0 mmol/L, base excess −10.9 mmol/L, and metHb 1.1%. Laboratory tests showed: creatinine 1.6 mg/dL, glucose 55 mg/dL, C-reactive protein (CRP) 4.9 mg/L, creatine kinase (CK) 3800 IU/L (Table 1). Over the next 2 hours she developed a severe shock state necessitating initiation of norepinephrine infusion. Meanwhile, serum lactate levels increased to 8.5 mmol/L and the patient exhibited diffuse bleeding in the throat, on puncture sites, the bladder probe but also from her rectum. Initial therapy consisted of fluid administration (that is 6000 mL crystalloids within 3 hours), intravenous glucose, and norepinephrine at 0.4 µg/kg/minute; also, fresh frozen plasma, prothrombin complex concentrates, tranexamic acid, and fibrinogen were given. At that point, the patient had hepatic failure, distributive shock, hypoglycemia, acute kidney injury, rhabdomyolysis, and severe acute respiratory distress syndrome (ARDS).

**Table 1.** Results from main biological tests

	Admission to other hospital	Admission to ICU	12 hours after ICU admission
White blood cells, n/mm^3^ (NV = 4000–1000)	6700	11,000	12,845
Hemoglobin, g/dL (NV = 13–16)	12.5	11.8	11.9
Platelets, n/mm^3^ * 10^3^ (NV = 150–450)	359	184	103
C-reactive protein, mg/dL (NV < 10)	4.9	128.0	213.5
Urea, mg/dL (NV < 40)	34	54	98
Creatinine, mg/dL (NV < 1.2)	1.6	2.5	2.2
Total bilirubin, mg/dL (NV < 1.2)	0.8	1.3	1.8
LDH, IU/L (NV < 250)	219	459	678
AST, IU/L (NV < 50)	78	289	1449
ALT, IU/L (NV < 50)	45	167	651
GGT, IU/L (NV < 35)	118	210	666
AP, IU/L (NV < 35)	89	178	176
CK, IU/L (NV <80)	3800	5100	8700
Glucose, mg/dL (NV = 80–115)	55	89	134
Lactate, mmol/L (NV < 2.0)	3.0	5.5	6.7

*LDH* lactate dehydrogenase, *AST* aspartate aminotransferase, *ALT* alanine aminotransferase, *GGT* gamma-glutamyltranspeptidase, *AP* alkaline phosphatases, *CK* creatinine kinases, *NV* normal values

**Fig. 1:**
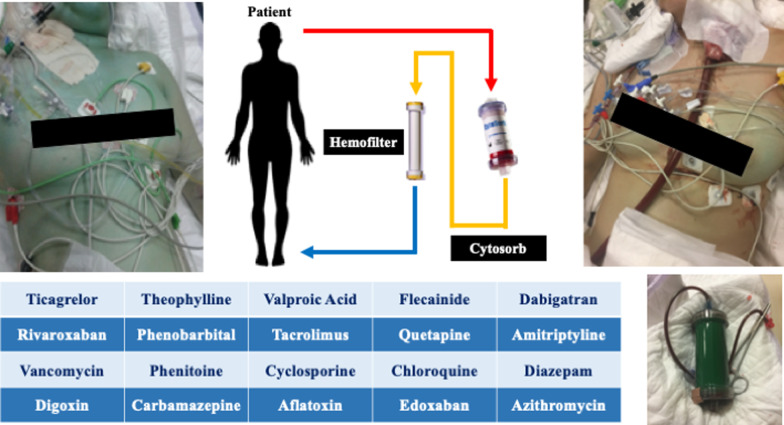
Changes in skin color of the patient and appearance of the first CytoSorb adsorber after treatment (green color due to extensive patent blue adsorption). The patient gave her consent for publication of images. A representation of the configuration for continuous renal replacement therapy with use of CytoSorb is also presented. At the bottom of the figure, we report a list of drugs that can potentially be removed by CytoSorb filters.

**Fig. 2:**
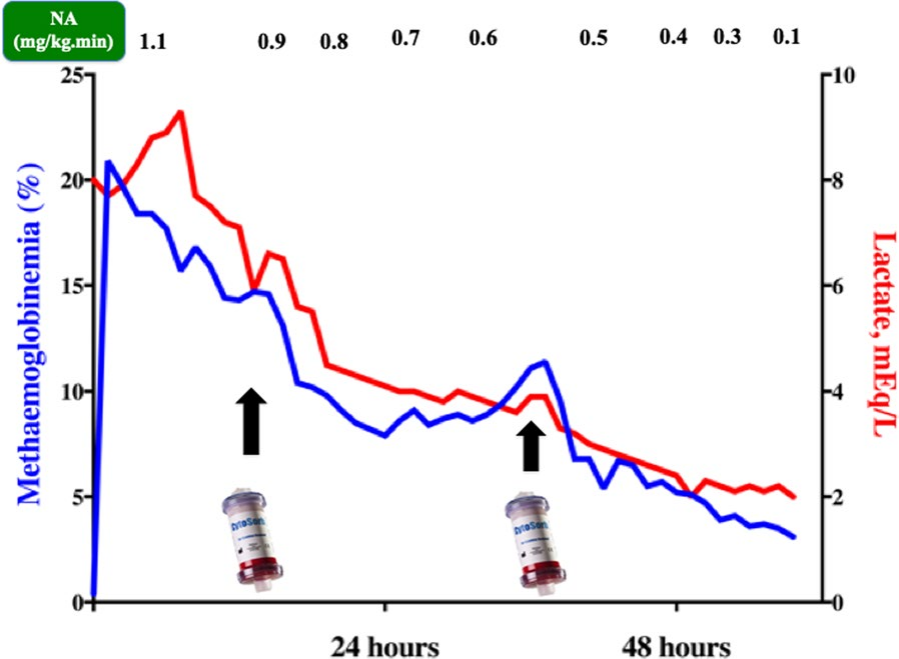
Time course of lactate and methemoglobin after initiation of CytoSorb therapy (arrows indicate absorber insertion).

Due to the option for potential liver transplantation, the patient was transferred to the Department of Intensive Care at Erasme Hospital, Brussels. On admission, she exhibited severe ARDS (pH 7.19, PaCO_2_ 33 mmHg, PaO_2_ 72 mmHg on FiO_2_ 100%, lactate 5.5 mmol/L, and metHb 1.2%), hemodynamic instability (that is, requirement for vasopressors), and persistent capillary leakage. Ongoing therapies included: propofol (2.0 mg/kg hour via continuous intravenous infusion—ivc), sufentanil (0.2 μg/kg hour ivc), norepinephrine (1.3 µg/kg/minute ivc), pantoprazole (20 mg iv q24h), cisatracurium (0.03 mg/kg ivc), and *N*-acetylcysteine (100 mg/kg day ivc). Results of blood samples are presented in Table 1. As a consequence, antibiotic therapy with amoxicillin/clavulanic acid (2 g q8h) and continuous renal replacement therapy (CRRT) was initiated; the device was set in hemodiafiltration mode with a blood flow rate of 160 mL/minute, a total CRRT dose (that is cumulative ultrafiltration and dialysate flows) of 55 mL/kg hour, and citrate anticoagulation. Additionally, administration of IV methylene blue (2 mg/kg one dose) was prescribed to reduce vasoplegia and to reduce the norepinephrine requirement. Nevertheless, immediately after dye injection, the skin of the patient became green/blue, oxygen saturation (SpO_2_) fell to 75%, and blood analysis revealed MetHb of 21% and lactate of 8.1 mmol/L, indicating severe tissue hypoxia. While the patient was rapidly treated with a veno-venous extracorporeal membrane (ECMO) device, it was discovered that Patent Blue V had been erroneously administered instead of methylene blue (both drugs being one beside the other in the pharmacy), resulting in life-threatening methemoglobinemia. As no significant improvement was observed on SpO_2_ and persistent metHb > 15% was observed after a few hours of ECMO and CRRT therapy, a CytoSorb adsorber (CytoSorbents Europe GmbH, Berlin, Germany) was installed into the extracorporeal CRRT circuit before the hemofilter (Fig. 1) and CRRT prescription remained unchanged. After 15 hours, the adsorber was changed and a second absorber was used for another 20 hours. This treatment resulted in a progressive and significant reduction in plasma metHb accompanied by a decrease in norepinephrine dose and of lactate levels (Fig. 2); removal of toxic Patent Blue V into the Cytosorb cartridge was also observed (Fig. 1). Analysis of urines (that is blood, proteins, and sediment), different serological tests (that is herpes virus, different hepatitis viruses, human immunodeficiency virus, *Borrelia burgdorferi*, *Treponema pallidum*, *Coxiella burnetii*, and *Rickettsia* spp) and microbiological samples (that is, blood cultures, tracheal aspirates, bronchoalveolar lavage, and urines) all tested negative. The follow-up period after cessation of CytoSorb treatment consisted of weaning from ECMO on day 5, from CRRT on day 7, tracheostomy on day 19, and weaning from mechanical ventilation on day 26. The patient was transferred to the ward on day 30 and was successfully weaned from tracheostomy on day 41, before being discharged to a rehabilitation facility on day 56 and finally back home on day 75. At 2 years after ICU admission, the patient presented full physical recovery and is dialysis independent. Since this event, methylene blue has been placed into the ICU pharmacy and Patent Blue V into the general hospital pharmacy.

## Discussion and conclusions

In the present case report, we successfully treated a 27-year-old woman exhibiting severe intoxication with methamphetamine and iatrogenic intoxication with Patent Blue V associated with multiple organ failure, using a combination of ECMO, CRRT, and CytoSorb hemoadsorption. Treatment was associated with a rapid and significant reduction in plasma metHb accompanied by improved hemodynamics and normalization of serum lactate levels. Moreover, treatment contributed to removal of circulating Patent Blue V.

As with any suspected drug ingestion, it is mandatory to obtain a detailed history to guide therapeutic interventions, including the type of drug, amount ingested, last ingestion time, frequency of usage, route, and co-ingested substances. However, this is often not possible due to the nature of the intoxication. Of note, MDMA is mostly manufactured illegally, thus its exact composition and purity are often difficult to determine. Importantly, it is not recommended to delay care before the identification of the causative substance or substances. Drug screening may be inaccurate or time-consuming, thus the recognition of a specific “toxidrome,” that is common symptoms suggesting potential intoxication from a group of different drugs, should result in urgent medical treatment to support failing organs. In our case, we initiated supportive care immediately after hospital admission, although different extracorporeal interventions were necessary to help the multiple organ dysfunctions.

The development of serious adverse effects from MDMA intoxication vary from individual to individual, with no established correlation between the amount of MDMA ingestion and the severity of the clinical manifestation, which is why individuals can die after consuming only a small dose of MDMA while others have survived despite consuming lethal doses [9]. As mentioned, the user may present with a wide variety of mild to life-threatening complications; there is no antidote for MDMA intoxication, so treatment options remain limited to symptomatic interventions. The patient described herein exhibited severe hemodynamic instability and hyperlactatemia. As the CytoSorb hemoadsorption device has been described as having beneficial effects on hemodynamics as well as metabolic function (that is, metabolic acidosis), we applied this technique with the intention of reducing toxic Patent Blue V plasma concentrations. During the course of the two treatments, norepinephrine requirements could be decreased significantly, which was accompanied by a resolution of the metabolic acidosis. These findings are in line with several case reports and series in critically ill patients treated with CytoSorb, although most of them were related to infectious complications [10–12]. The extent to which Patent Blue V was adsorbed by the CytoSorb device remains questionable, as it was not measured during the course of this epicrisis. However, Patent Blue has an average molecular weight that should theoretically fit the adsorption spectrum of the device (that is, removal of “toxins” with molecular weights ranging from 5 to 60 kDa). Furthermore, the physical appearance of the cartridge suggested that considerable amounts of the substance had been removed from the bloodstream. As such, one can argue that progressive removal of Patent Blue V by the cartridge led to a point where the endogenous reduced nicotinamide adenine dinucleotide (NADH)-dependent enzyme methemoglobin reductase, which is responsible for converting methemoglobin back to hemoglobin, was able to convert most of residual metHb level and reverse the detrimental process.

This is the first case report describing the combined application of multiple extracorporeal support combined with CytoSorb hemoadsorption in a patient with MDMA intoxication accompanied by iatrogenic intoxication with Patent Blue. Neither MDMA nor Patent Blue have any known antidotes; therefore, their rapid and effective removal by the CytoSorb cartridge based on clinical parameters offers a unique opportunity in such cases of acute intoxication. There is a potential role for CytoSorb in wider drug intoxications, but this needs to be verified in larger series. A list of drugs that can be removed by CytoSorb is presented in Fig. 2.

## Data Availability

Data are available on request to the authors.

## Abbreviations

ARDS: Acute respiratory distress syndrome
CRRT: Continuous renal replacement therapy
DIC: Disseminated intravascular coagulation
MetHb: Methemoglobin
MDMA: 3.4-Methylenedioxymethamphetamine
SpO_2_: Oxygen saturation
V-V ECMO: Veno-venous extracorporeal membrane oxygenation
